# The link between the trans-Golgi network and tumour progression

**DOI:** 10.1007/s11033-025-10548-6

**Published:** 2025-04-28

**Authors:** Leila Jahangiri

**Affiliations:** 1 School of Science and Technology, Nottingham Trent University, Clifton Site, Nottingham, NG11 8NS UK; 2 Division of Cellular and Molecular Pathology, Department of Pathology, Addenbrookes Hospital, University of Cambridge, Cambridge, CB0 2QQ UK

**Keywords:** Trans-Golgi network, Endosomes, Tumour, Progression, Trafficking

## Abstract

The trans-Golgi network is a major sorting organelle consisting of a tubular membrane originating from the trans-Golgi cisternae. Proteins and lipids synthesised in the endoplasmic reticulum are transported through the Golgi apparatus and sorted in the trans-Golgi network into pleomorphic transport carriers targeted for various destinations. These destinations include the apical and basolateral membranes, early and late, recycling endosomes, and secretory granules. The trans-Golgi network also accepts retrograde endosome traffic, contributing to the recycling of proteins and lipids, and, therefore, sits at the crossroads of secretory and endosomal systems. Cancer is a somatic evolutionary process that comprises the accumulation of mutations that contribute to tumourigenesis, growth, progression, immune evasion, and resistance to therapy. This study aims to catalogue how multiple components and players of the trans-Golgi network affect tumour progression. Further, the link between the tumour microenvironment, the trans-Golgi network, and tumour progression will be dissected. A more profound understanding of these mechanisms will inform better treatment options.

## The trans-Golgi network and tumour progression

### Introduction to the trans-Golgi network

**Fig. 1:**
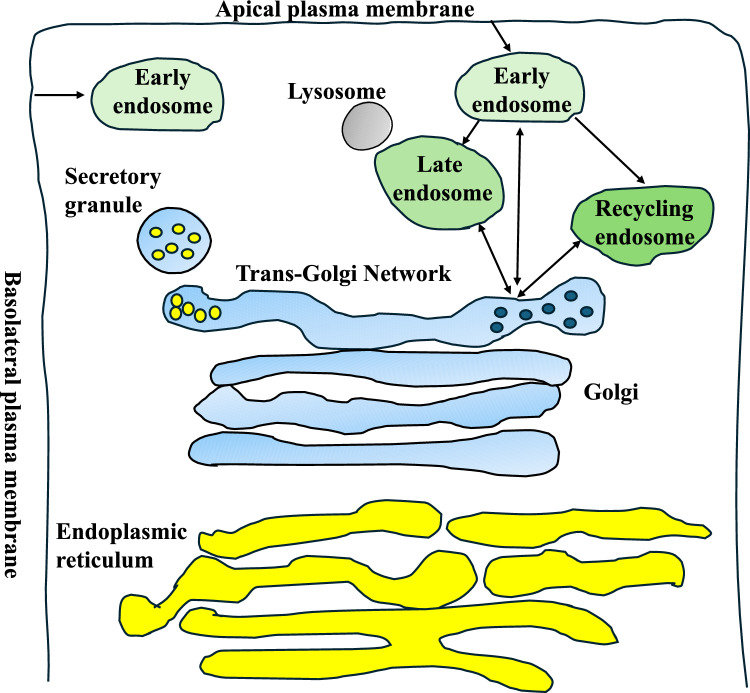
Sorting across endocytic and exocytic pathways by the TGN in a typical epithelial cell. The TGN originates from the trans-Golgi cisternae, and proteins synthesised in the ER proceed through the Golgi and are directed to multiple destinations (i.e., the basolateral or apical plasma membranes and specialised secretory granules, in addition to early, late, and recycling endosomes). The exit of cargo through the membranes is not shown in the figure. The TGN receives input from endocytosis pathways and sends cargo back to other Golgi components (these are represented by double-ended arrows that signify cargo entering/exiting the endosomes from/to the TGN). Early endosomes are formed from the apical and basolateral plasma membranes. The yellow and blue tubules represent the ER and the Golgi apparatus, respectively. The early, late and recycling endosomes were shown using shades of green. All figures were generated by the author using Microsoft PowerPoint. (Color figure online)

The newly synthesised secretory pathway proteins that do not remain in the endoplasmic reticulum (ER) pass through the Golgi apparatus, which consists of a series of flattened membrane-bound cisternae. These cisternae are usually found in stacks associated with vesicles [1]. Notably, the Golgi apparatus consists of the cis, medial, and trans sections, and the proteins synthesised, glycosylated, folded, and checked in the ER will enter the cis face of the Golgi by getting loaded into vesicular carriers. Then they proceed through the Golgi stacks and subsequently to the trans-Golgi network (TGN) [2]. On that note, two tubular-reticular networks can be defined in the Golgi: the cis-Golgi networks (CGN) or the ER–Golgi intermediate compartment (ERGIC), and the TGN. The CGN/ERGIC are the entry site for ER cargo and could be formed from the ER-derived transport vesicles (vesicular tubular complex/structure: VTC/S). The TGN, however, is defined as a specialised organelle located on the trans side of the Golgi that sorts, collects, and packages newly synthesised proteins to their destination. These destinations include early and late endosomes, recycling endosomes, apical and basolateral plasma membranes, or secretory vesicles [2]. In mammalian cells, routes of endocytic trafficking and recycling include early and sorting endosomes, while delayed recycling can occur through the recycling endosomes [3]. In addition to the sorting and secretory cargo transport and delivery, the TGN accepts endosomal traffic as well (usually proteins from its sorting system that are now recycled back from the plasma membrane in addition to cholesterol and sphingolipid cargo); hence, it is an interface between exocytosis/secretion and the endocytosis systems [2, 4] (Fig. 1). The TGN also utilises various Golgi proteins (such as Golgi membrane protein 1 (GOLM1)), docking, and fusion factors such as soluble N-ethylmaleimide sensitive factor attachment protein receptors (SNAREs), Rab GTPases, and motor and cytoskeleton proteins that assist it with these processes [1, 5]. Another aspect of TGN function is post-translational modifications of proteins and transfer, insertion, and completion of lipid biosynthesis [2]. Having examined the TGN in 14 mammalian cell types, this organelle was shown to be neither stable nor permanent but dynamic. In cells with large secretory granules, the TGN was lacking; in cells with small- or medium-range secretory granules, small TGNs exist. Finally, cells with extensive lysosomal systems (but no large secretory granules) displayed multilayered TGNs [6]. Other early studies by Griffiths established the size of the TGN and the Golgi in baby hamster kidney cells under various conditions, including different temperatures [7]. For example, at 20° centigrade, the size of the Golgi stacks before the TGN was smaller while the TGN was larger, suggesting that the TGN and Golgi are highly dynamic structures [7]. In more recent years, evidence has offered more insight into the function of the TGN. For example, the TGN could be an independent organelle. In plant cells, the TGN can be positioned away from the Golgi, substantiating the existence of a Golgi-independent TGN alongside a TGN associated with the Golgi [8, 9]. Studies in yeast have suggested the existence of two compartments in the TGN, including the early and late TGN. The former acts as a cargo reception centre, and the latter forms carriers such as clathrin, GGA (a Golgi protein), and clathrin adaptor complex-1 (AP-1) [10] (reviewed extensively in [11]).

### Introduction to cancers and tumour progression

Cancers have devastating effects on patients globally and display diverse and varied genetic profiles [12]. Cancer is a multistage evolutionary process predicated on transforming the cell of origin through acquiring a host of genetic alterations that confer proliferation, growth, and survival advantages. These advantages will contribute to the process of tumourigenesis [13, 14]. Although cancer is a complex disease whose pathology cannot be understood by studying isolated proteins, genes, pathways, and mechanisms, it is vital to understand these aspects in depth before building an integrative picture [15]. Tumours constantly crosstalk with their microenvironment (the TME), which comprises extracellular matrices composed of glycoproteins, proteoglycans, and proteins that undergo degradation and reconstitution, relaying messages and signals to the tumour cell [16, 17]. This study aims to 1—examine how various TGN-associated proteins and components contribute to tumour progression and 2—assess the link between the TME, and the TGN and tumour progression.

## TGN-associated proteins and other partners at the crossroads of malignancy

### Rab GTPases

**Fig. 2:**
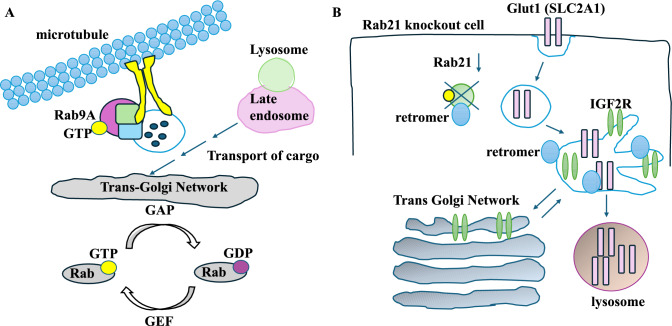
Rab proteins, the TGN, and tumour progression. **A** Rab9A, a Rab GTPase, interacts with microtubules and other proteins that transport cargo from the late endosomes to the TGN. Rab9A influenced cancer invasion and migration. Rabs obtain a GTP by guanine nucleotide exchange factor (GEF), while GTPase activating protein (GAP) activates Rab’s GTPase function. **B** Rab21 GTPase cooperated with the retromer complex on retrograde trafficking. Rab21 knockout reduced endosome-to-plasma membrane recycling of the SLC2A1 glucose transporter (Glut1). It is then degraded in the lysosomes. IGF2R trafficking between the TGN and endosome increased, and so did the enzymatic load of the lysosome

Rab9, a Rab GTPase, transports mannose-6-phosphate receptor (involved in the retrograde transport and delivery of lysosomal enzymes) from the late endosomes to the TGN and has roles in endosome dynamics [18]. Rab9 interacted with the p40 protein (involved in endosome-to-TGN transport) and together directed the docking of transport vesicles [18, 19]. This study focused on the Rab9A isoform in liver cancer cell lines Hep3B and HepG2. The overexpression of Rab9A enhanced proliferation in the Hep3B cell line, while its knockdown in the HepG2 cell line reduced it. The overexpression of Rab9A suppressed apoptosis in Hep3B cells by reducing Bax and cleaved caspase 3 and increasing BCL-2 levels (three proteins associated with apoptosis). Rab9A overexpression increased the migration of Hep3B cells. Mechanistically, Rab9A acted through AKT/mTOR signalling since BEZ235 (an inhibitor of AKT and mTOR phosphorylation) suppressed Rab9A-induced proliferation and invasion [18]. Overall, Rab9A affected tumour progression through the AKT/mTOR pathway (Fig. 2A). Another study focused on Rab9 in human breast cancer MDA-MB-231 and MCF7 cell lines. Rab9 overexpression increased proliferation and migration in these cell lines. In Rab9 knockdown MDA-MB-231 and MCF7 cell lines, a higher Bax/BCL-2 ratio was detected, marking increased apoptosis [20]. Mechanistically, in Rab9 knockdown MDA-MB-231 and MCF7 cell lines, the phosphorylation of p-AKT and p-mTOR was decreased. Overall, the authors showed that Rab9, a GTPase involved in the retrograde transport of cargo to the TGN, relied on signalling pathways to promote tumour progression [20].

Other proteins, such as Rab42, regulated the movement of vesicles from the endosomes to the TGN. The expression of Rab42 was linked to an increased cell infiltration of M2 macrophages and Treg cells to the tumour microenvironment and immune checkpoint molecules [21]. The knockdown of Rab42 decreased proliferation, migration, and invasion in MHCC-97H hepatocellular carcinoma (HCC) cells [21]. In another study, the effect of Rab42 on programmed death-ligand 1 (PD-L1) expression and immune tolerance was assessed. Silencing Rab42 using siRNA (siRab42) reduced cell viability and proliferation and boosted apoptosis. It reduced PD-L1 expression in MHCC-97H cells [22]. Mechanistically, co-transfecting siRab42 and E2F1 plasmids in MHCC-97H cells rescued the effect of siRab42 on proliferation, apoptosis, and PD-L1 expression. Therefore, Rab42 exerted its effects through E2F1 [22]. Rab42 affected proliferation and immune tolerance through the E2F1 pathway or the infiltration of immune cells.

The endosomal system, the retromer complex (VPS35-VPS29-VPS26 proteins), and Rab21 regulate retrograde vesicle trafficking and protein recycling from endosomes to the TGN [23]. Cargo specificity of retromer complexes is established by sorting nexin proteins [24]. Rab21 co-immunoprecipitated with VPS35, suggesting the link between Rab21 and retrograde trafficking. Mechanistically, Rab21 knockout (KO) in HeLa cells led to the retromer-associated recycling of SLC2A1 to the intracellular vesicles. LAMP1, a marker of lysosomes, overlapped with SLC2A1, suggesting its hydrolysis by the lysosome. This reduced cell surface SLC2A1 and glucose uptake. This also contrasted with the plasma membrane localisation of SLC2A1 in control cells [23]. Retrograde trafficking of IGF2R between the endosomes and the TGN was also increased in Rab21 KO cells. IGF2R usually transports enzymes to the lysosome, and in Rab21 KO cells, IGF2R increased their enzymatic load. Rab21 KO MDA-MB-231 cells formed smaller tumours than controls in vivo. Overall, Rab21 promoted endosomal recycling, cellular energetics, and tumour progression [23] (Fig. 2B).

To summarise the studies, it should be noted that Rab9 interacted with p40 to facilitate endosome-to-TGN transport; it promoted proliferation and migration through the AKT/mTOR pathways [18, 20]. Rab42 promoted tumour proliferation, and migration and triggered immunotolerance [21]. It also promoted proliferation and PD-L1 expression in HCC cells by regulating the E2F1 pathway [22]. Finally, Rab21 cooperated with the retromer complex for the retrograde trafficking of SLC2A1 and IGF2R and promoted tumour proliferation [23].

### Golgi proteins

**Fig. 3:**
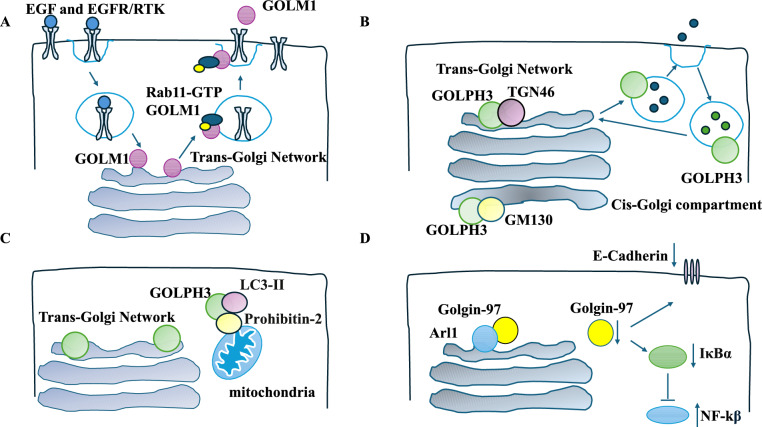
Golgi proteins, the TGN, and tumour progression. **A** GOLM1 was recruited to EGFR/RTK once internalised. EGFR colocalised with Rab11 after EGF stimulation. GOLM1 recruited Rab11 and EGFR/RTK into a complex, enabling the return of the receptors to the surface. **B** GOLPH3 overlapped with two Golgi compartments: the cis-Golgi (GM130 marker) or the TGN (TGN-46 marker). GOLPH3 protein emerged from the TGN and VTS to the periphery and from the periphery to the Golgi. **C** GOLPH3 bound prohibitin-2 and LC3-II and, thereby, activated autophagy. **D** Golgin-97 tethered to the TGN via Arl1. Golgin-97 knockdown induced NF-κB by reducing IκBα levels. E-cadherin was downregulated

GOLM1 is a Golgi-anchored membrane protein [25]. GOLM1 was high in the GOLM1^high^ invasive MHCC-97H cell line and lower in the GOLM1^low^ non-invasive Hep3B cell line. GOLM1 knockdown reduced proliferation and migration levels in GOLM1^high^ cells more effectively than in GOLM1^low^ cells. This was confirmed in MHCC-97H^shGOLM1^xenograft mouse models which generated smaller tumours than controls [25]. In MHCC-97H cells, GOLM1 colocalised with TGN-46 protein. Epidermal growth factor (EGF) exposure led to the transport of GOLM1 proteins from the TGN to the cytoplasm, where it colocalised with Rab5 + vesicles and internalised EGFR/receptor tyrosine kinase (RTK). Live imaging showed that interactions between internalised EGFR/RTK and Rab5 + GOLM1 vesicles peaked around 30 min after EGF exposure [25]. Rab11 is a well-established recycling compartment-based GTPase involved in EGFR recycling. In non-targeting shRNA MHCC-97H cells, EGFR colocalised with Rab11 after EGF stimulation, in contrast to MHCC-97H^shGOLM1^ cells, in which EGFR showed lysosomal localisation. The authors also showed evidence of GOLM1 and Rab11 physical interaction that returned the receptors to the cell surface. Mechanistically, GOLM1-expressing cells increased EGFR recycling and cell migration and invasion [25] (Fig. 3A). Overall, GOLM1 recruitment of Rab11 and EGFR/RTK led to EGFR-mediated signalling and tumour migration.

Golgi phosphoprotein 3 (GOLPH3), a peripheral membrane-bound protein, was linked to vesicles and tubules of the Golgi [26]. The study examined the properties of GOLPH3 in breast cancer cell lines, MCF7 and MDA-MB-231, and a non-cancerous MC710A. Electron and fluorescent microscopy of all three cell lines showed that GOLPH3 localised to two Golgi compartments: the cis-Golgi (colocalisation with the GM130 marker) or the TGN (colocalisation with the TGN-46 marker) [26]. Brefeldin A treatment (which suppressed vesicle formation) led to the redistribution of GOLPH3 to the microtubule-organising centres in MCF7 cells and the cytosol or ER in the other 2 cell lines [26]. Tracking GFP-GOLPH3 protein in all three cell lines showed that it emerged from the Golgi, while in MCF7 cells, the VTS moved to the periphery and from the periphery to the Golgi (retrograde trafficking), showing distinct GOLPH3 membrane association characteristics [26] (Fig. 3B).

GOLPH3 was also associated with autophagy. Microtubule-associated protein 1 light chain 3 (LC3-II) levels were decreased in GOLPH3 knockdown U251 glioblastoma cells treated with or without rapamycin (an autophagy inducer). The overexpression of GOLPH3 had the opposite effect [27]. Electron microscopy showed that GOLPH3 downregulation suppressed autophagosomes and autolysosomes, suggesting GOLPH3 triggered autophagy. GOLPH3 overexpression promoted cell growth via triggering autophagy since inhibiting autophagy using chloroquine hindered this stimulatory effect on cell growth (mirrored in xenograft mouse models transplanted with U87 cells and treated with chloroquine) [27]. Mechanistically, GOLPH3 recruited inhibitin-2 (a mitochondria autophagy regulator) and LC3-II [27]. GOLPH3 mediated LC3-II levels through prohibitin-2 since prohibitin-2 knockdown reduced LC3-II levels in GOLPH3 overexpressing cells. Co-immunoprecipitation showed that GOLPH3 acted as a scaffold to recruit LC3-II and prohibitin-2 to trigger autophagy and tumour progression [27, 28] (Fig. 3C). GOLPH3 promoted tumour progression.

GOLPH3 was also expressed in T98G glioblastoma multiforme cells and regulated EGFR trafficking. The knockdown of GOLPH3 in T98G cell lines lowered proliferation rates and increased total EGFR levels and cell surface EGFR. The post-translational modifications of EGFR, such as sialylation of N-glycans and fucosylation, decreased in these cells [29]. In GOLPH3 knockdown cells, EGFR was localised to the cell surface and protrusions. EGFR was fused to EGFP and streptavidin-binding protein (SBP) to form SBP-EGFP-EGFR. This was co-expressed with streptavidin-KDEL retention peptide in wildtype and GOLPH3 knockdown cells. Exposure to biotin allowed it to compete with SBP for streptavidin and release SBP-EGFP-EGFR from the ER (RUSH assay) [29]. SBP-EGFP-EGFR also showed enrichment in cell protrusion regions in GOLPH3 knockdown cells. The authors showed that EGFR internalisation, recycling and post-translational modifications were affected in GOLPH3 knockdown cells [29]. GOLPH3 regulated EGFR recycling and tumour progression.

Golgin-97 tethers to the TGN via the Arf-like protein 1 (Arl1, a small GTPase) and promotes vesicle trafficking [30]. siRNA-mediated golgin-97 knockdown increased migration in MDA-MB-231 cells. This knockdown triggered the expression of NF-κB target genes such as adhesion molecules, metalloproteases, and cytokines. Interestingly, inhibiting NF-κB in golgin-97 knockdown MDA-MB-231 cells reduced migration. Mechanistically, golgin-97 knockdown induced NF-κB by reducing IκBα levels (IκBα inhibitor family). The golgin-97 knockdown activation of NF-κB was not linked to Golgi fragmentation. Golgin-97 knockdown cells showed reduced E-cadherin (and increased cell motility) [30]. Overall, golgin-97 downregulation induced tumour progression, motility and invasion (Fig. 3D). Further, Golgi fragmentation due to chemotherapy was studied in colorectal cancer. *miRNA-3135b* bound the 3′-untranslated region of GOLPH3, and the combination of chemotherapy and *miRNA-3135b* reduced chemotherapy-induced Golgi fragmentation [31].

Golgin-97 KO MDA-MB-231 breast cancer cells showed increased metastasis. Golgin-97 KO in zebrafish and xenograft mouse models also promoted tumour progression [32]. The application of multi-omics methods identified signalling cascades such as MAPK kinase, Wnt signalling, and inflammatory pathways as culprits in mediating the tumour progression effect of Golgin-97 KO. Accordingly, suppressing ERK1/2 and p38 MAPK reduced tumour progression and migration. For example, Golgin-97 KO cells treated with SB203580 (p38 MAPK inhibitor) and U0126 (MEK1/2 inhibitor) as single or double agents reduced Golgin-97 KO-induced cell migration. In xenograft mouse models, these compounds reduced inflammation and tumour progression at the primary tumour site [32].

To summarise the studies, it should be noted that GOLM1 mediated the recruitment of Rab11 and EGFR to allow for the return of the receptor to the cell surface and promoted tumour progression [25]. GOLPH3 emerged from the TGN and was involved in the retrograde trafficking of cargo and thereby could affect tumour signalling [26]. GOLPH3 promoted tumour growth by triggering autophagy [27]. GOLPH3 also affected EGFR recycling and post-translational modifications [29]. Golgin-97 was a tumour suppressor [30, 32].

### Other Golgi proteins

Other proteins may play roles. GRIP and coiled-coil protein 2 (GCC2) affected non-small cell lung cancer since its knockdown in the H640 cell line reduced cell viability and increased apoptosis. In GCC2 knockdown cell lines, migration and invasion were reduced [33]. In the xenograft mouse model, GCC2 knockdown H460 cells formed smaller tumours than controls; hence, GCC2 promoted tumour growth. GCC2 knockdown suppressed GCC2 + exosome secretion (thereby lowering their colony formation effect). GCC2 knockdown H460 cells showed reduced EGFR/RTK and MAPK/ERK pathway genes, suggesting the involvement of signalling pathways. Mechanistically, GCC2 colocalised with GM130, and GCC2 knockdown in H460 and H1299 cell lines led to the loss of Golgi integrity. GCC2 knockdown reduced vesicle trafficking of EGFR to the nucleus and tumour proliferation [33]. Overall, GCC2 knockdown affected EGFR-induced proliferative signals.

Syntaxin 6 is a target SNARE located in the TGN and endosomes. Syntaxin 6 was expressed in HCC tissue and was associated with advanced histological grades [34]. Syntaxin 6 overexpression in Huh7 and HepG2 cells promoted migration and proliferation (proliferating cell nuclear antigen, PCNA, and cyclin D1 levels increased). Using in vivo models, shSyntaxin 6 knockdown HepG2 cells xenografted to BALB/c nude mice formed small tumours (with low PCNA and Ki-67 levels) compared to controls [34]. Mechanistically, co-immunoprecipitation of a Flag-tagged syntaxin 6 showed binding to Rack1 and the binding between RACK1 and STAT3 (the associated signalling of syntaxin 6) [34]. Syntaxin 6 induced tumour progression through an adaptor, RACK1, which recruited STAT3.

The six-transmembrane protein of the prostate (STAMP1) was present in the TGN [35]. This gene was expressed in androgen-sensitive LNCaP prostate cancer cell lines in the presence or absence of synthetic androgen (R1881). Androgen-dependent prostate cancer CWR22 xenograft tumours (grown in mice fed with testosterone pellets) displayed low STAMP1 expression compared to their relapsed derivatives (CWR22R tumours), suggesting STAMP1 was indicated in tumour progression and relapse. GFP-STAMP1 in COS-1 cells was localised with TGN-46, the VTS, and the plasma membrane [35]. Time-lapse microscopy showed the to-and-from trafficking of GFP-STAMP1 from the TGN via the VTS. STAMP1 was linked to tumour progression and relapse [35].

To summarise these studies, it should be noted that GCC2 promoted tumour growth by modulating vesicle trafficking of EGFR to the nucleus and sustaining the integrity of the Golgi [33]. Syntaxin 6 promoted proliferation and invasion in HCC cell lines through the RACK1/STAT3 axis [34]. STAMP1 was linked to prostate cancer relapse [35]. TGN-related proteins and effectors impacted tumour progression by modulating signalling pathways.

### Clathrin adaptor and pumps

**Fig. 4:**
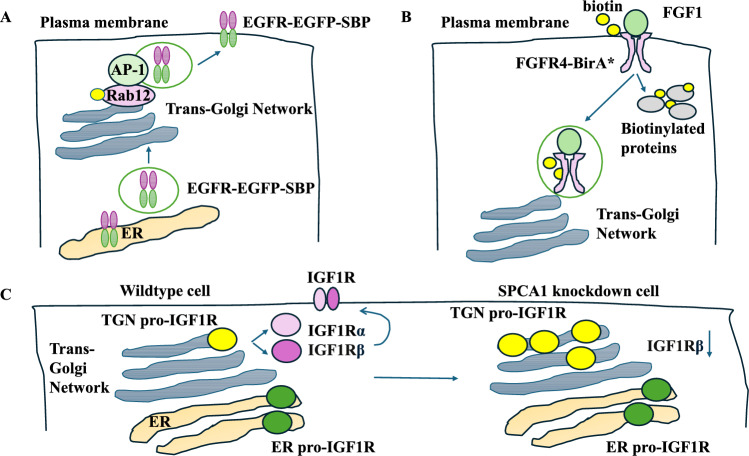
AP-1, pumps, the TGN, and tumour progression. **A** AP-1 and Rab12 regulated the TGN export of EGFR to the cell membrane and EGFR-related cellular functions. **B** FGF1 bound to and activated FGFR4-BirA*. Upon ligand binding and receptor activation, the protein complex was internalised as an endosome, and nearby proteins were biotinylated. **C** There was a decrease in IGF1Rβ production along with an accumulation of TGN pro-IGF1R in SPCA1 knockdown cells

AP-1 and Rab12 affected the transport of EGFR from the TGN to the cell membrane. The study used a RUSH assay to test if AP-1 regulated the TGN export of EGFR. In this study, the trafficking of SBP-EGFP-EGFR protein was investigated in the AP-1 gamma component KO HeLa cells (AP1-γ1 KO) and controls [36]. The AP1-γ1 KO cells showed much higher cytoplasmic accumulation of SBP-EGFP-EGFR than controls. The expression of the AP1-γ1-HA tag reduced the cytoplasmic accumulation of the protein in AP1-γ1 KO cells. Rab12 knockdown was also important in the TGN export of EGFR since Rab12 knockdown in HeLa cells led to the juxtanuclear accumulation of SBP-EGFP-EGFR protein [36]. AP-1 and Rab12 proteins were crucial for cell morphology (elongation), and reduced proliferation was observed in AP1-γ1 KO or Rab12 knockdown HeLa cells. Surface labelling showed a reduction in the cell-membrane localisation of endogenous EGFR in AP1-γ1 KO or Rab12 knockdown cells. AP-1 and Rab12 regulated the post-Golgi transport of EGFR to the surface [36] (Fig. 4A). Finally, AP1-γ1 promoted tumour progression in breast cancer since its depletion reduced growth and migration [37].

A study focused on the retrograde trafficking and fibroblast growth factor receptor (FGFR) from internalisation by clathrin to transport through endosomes to the TGN. FGFR4 was fused with a mutated biotin ligase (BirA*) to form FGFR4-BirA* protein in U2OS cells [38]. Fluorophore-labelled FGF1 (DL550-FGF1) bound to FGFR4-BirA* and activated it. Upon binding and activation, the protein complex was internalised, and proteins close to FGFR4-BirA* were tagged with biotin. The cells with activated receptors (FGFR4-BirA* with FGF1) were subjected to mass spectrometry, showing the enrichment of FGFR signalling pathway proteins, NCK2, RSK2, and FRS2. Gene ontology terms including clathrin adaptor complex, clathrin coat, and recycling endosome were enriched [38]. Super-resolution microscopy showed that FGFR4-BirA* bound to FGF1 and colocalised at the cell surface, intracellular compartments, and endosomes. FGFR4 used the clathrin-based endocytosis route for internalisation. Mechanistically, the transport of labelled FGFR4/FGF1 in clathrin heavy chain (CHC)-depleted cells resulted in the cell surface accumulation of the FGFR4. This also affected the signalling cascade downstream of AKT and ERK [38] (Fig. 4B). Overall, clathrin affected FGFR signalling and tumour progression.

A study focused on a Golgi calcium pump, SPCA1, in the basal breast cancer subtype [39]. The knockdown of SPCA1 in the MDA-MB-231 breast cancer cell line reduced proliferation and induced a rounded cell morphology in these cells, suggesting links to tumour progression. The protein levels of plasma membrane ATPase and endoplasmic reticulum calcium ATPase (SERCA2) were not altered in SPCA1 knockdown COS-7 cells. The endoplasmic reticulum calcium modulation in SPCA1 knockdown cells was assessed by measuring [Ca2 +] _CYT_ levels and the area under the [Ca2 +] _CYT_ curve in the presence of the SERCA2 inhibitor (CPA) [39]. This showed a mild decrease in calcium peak and area under the curve, suggesting a mild effect of SPCA1 depletion on ER/CPA calcium dynamics. Unprocessed forms of insulin-like growth factor 1 receptor (IGF1R) comprised ER (ER pro-IGF1R) and TGN (TGN pro-IGF1R). The TGN pro-IGF1R is processed to IGF1Rα and IGF1Rβ, and they form receptors at the cell surface. Silencing SPCA1 in the MDA-MB-231 cell line led to a decrease in IGF1Rβ production and an increase of pro-IGF1R in the TGN. Overall, the depletion of a TGN calcium pump affected cell proliferation and IGF1R processing [39] (Fig. 4C). SPCA1 promoted tumour progression by affecting important growth factor receptors.

To summarise the studies, it should be noted that AP-1 and the SPCA1 pump affected tumour progression. Rab12 and AP-1 affected the cell membrane localisation of endogenous EGFR. These proteins were crucial for EGFR-related cellular functions such as proliferation [36]. FGFR4/FGF1 complex used the clathrin-based endocytosis route for internalisation, and its effects were mediated by AKT/ERK pathways, suggesting links to cancer signalling [38]. Silencing SPCA1 led to the accumulation of TGN pro-IGF1R and decreased IGF1Rβ [39].

## Changes in the TME alter TGN response and are linked to malignant transformation

The TME includes the extracellular matrix, comprising a meshwork of highly interconnected and dynamic proteins and glycoproteins. The TME also includes stromal and immune cells, and inflammatory and secreted molecules [16]. During tumourigenesis, the interaction between the TME and cancer cells can lead to stiffening of the extracellular matrix and changes to signal transduction [40]. Changes in the stiffness of the extracellular matrix can also lead to more aggressive phenotypes of cancer cells [40]. It is widely acknowledged that changes in Golgi products can alter the TME and impact tumour cell migration and vice versa [32]. For example, in cancers, altered Golgi and TGN functions can increase immunosuppressive cytokines, which promote tumour progression [41]. Therefore, understanding the changes induced in the TGN during tumourigenesis is important.

**Fig. 5:**
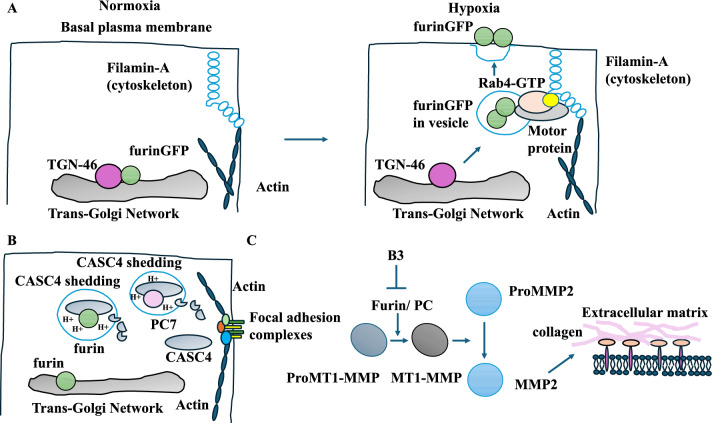
The tumour microenvironment, the TGN, and tumour progression. **A** Furin colocalised with TGN-46. Hypoxia triggered the re-localisation of furin from the TGN to the endosomes and the plasma membrane. Rab4 (GTP) and filamin-A assisted in the re-localisation of furin to the basal plasma membrane. **B** CASC4 was shed by furin/PC7 in endosomes or TGN with acidic conditions. CASC4 increased the focal adhesion complexes and promoted tumour migration. **C** B3 reduced solMT1-MMP maturation and the activity of MMP-2 on the extracellular matrix

One aspect that links the TME with tumour progression and the TGN is hypoxia [17, 42, 43]. Furin is a proprotein convertase in the TGN, Golgi, and the plasma membrane. Hypoxia promotes the relocalisation of furin from the TGN to the endosomes and the plasma membrane of cancer cells [44]. This was tested by expressing a furinGFP construct in HT-1080 fibrosarcoma cells, which showed furinGFP localised with TGN-46 in the perinuclear region under normoxic conditions [44]. Hypoxia decreased the colocalisation of furin and TGN-46. Instead, it led to the cytoplasmic dispersion of the furin-containing vesicles and furin localisation to the basal plasma membrane. The reversal of hypoxic conditions redistributed furin to the perinuclear TGN-46 locations [44]. Mechanistically, the overexpression of Rab4 under normoxic and hypoxic conditions increased the plasma membrane localisation of furin. Under hypoxic conditions, furin was redistributed around the filamin-A-rich areas (an anchoring cytoskeleton protein). Blocking the interaction between furin and filamin-A prevented the relocalisation of furin to the plasma membrane and periphery under hypoxia. Filamin-A-based furin tethering was essential for cell migration [44] (Fig. 5A).

Targets of furin may be significant. Cancer susceptibility candidate 4 (CASC4) had cleavage sites for furin/PC7 (proprotein convertases). Co-expression studies showed that PC7 shed CASC4 in HEK293 cells transfected with cDNA for CASC4 and PC7 [45]. Brefeldin A dramatically reduced CASC4 cleavage by PC7 and furin since this compound inhibited the transport of proteins from the ER to the Golgi [45]. Blocking COPII vesicle formation involved in cargo transport from the ER to the Golgi had a similar effect. CASC4 was shed by PC7 or furin in endosomes or the TGN with acidic environments since treating cells with an alkalinising agent (NH4Cl) reduced shedding. CASC4 knockdown in MDA-MB-231 breast cancer cells increased migration. Mechanistically, CASC4 overexpression increased the focal adhesion complexes but reduced the activity of Rho-GTPase Cdc42. CASC4 suppressed tumour migration and progression, while furin/PC7 cleaved CASC4 [45] (Fig. 5B). Notably, Na + /H + transporter 7 (NHE7) shuttled between the TGN, endosomes, and plasma membrane [46]. In MDA-MB-231 breast cancer cells, NHE7 overexpression increased cell–cell adhesion, cell invasion, and growth [46].

Furin/PC can indirectly target matrix metalloprotease 2 (MMP-2) and affect motility [47]. In Chinese hamster ovary (CHO) cells co-expressing a soluble form of MT1-MMP (pro/solMT1-MMP, a metalloprotease target of furin) and furin did not affect the processing of solMT1-MMP. In the presence of 15 uM of the B3 inhibitor (an inhibitor of furin/PC), solMT1-MMP processing and its mature form were reduced [47]. This decreased the activity of MMP-2 and cell motility in CHO cells. Furthermore, CHO cells expressing proMT1-MMP-HA activated MMP-2, which degraded gelatine and allowed the cells to migrate, whereas proMT1-MMP-HA expressing CHO cells exposed to 15 uM of B3 showed lower motility (due to the inability to activate MMP-2 and degrade gelatine). In invasive HT1080 human fibrosarcoma cells, B3 in the presence of ConA (which converted proMMP-2 to MMP-2) decreased MT1-MMP and the mature form of MMP-2 (and invasiveness) [47]. Furin/PC was linked to tumour progression via degrading extracellular matrix proteins (Fig. 5C).

As reviewed earlier in proteins of the TGN, GOLPH3 was shown to recruit prohibitin-2 and LC3-II to promote autophagy and tumour progression [27]. In a similar study, autophagy was implicated in organ wasting to provide the necessary nutrients for tumour growth in *Drosophila melanogaster* [48]. Accordingly, during tumour progression, heightened nutrient demands were met by increasing autophagy levels through the lysosomal degradation of proteins and organelles. Further, cancers driven by Ras could trigger autophagy through the TME to mobilise amino acids to the tumour to support tumour growth. This study showed that autophagy-driven organ wasting, and mobilisation of nutrients could also further support tumour growth. X-ray tomography assessed a *Ras*^*V12*^*, scrib-/-* mutant. The subjects formed eye tumours which eventually invaded the central nervous system. While tumours grew tenfold and invaded the neighbouring tissues between days 6–10, muscle volume decreased 50% in the same period. Also, in these mutants’ muscle and adipose tissue wastage preceded reduced food consumption and mobility, suggesting the independence of tissue wastage. The underlying mechanism for organ wastage was systemic autophagy activation [48]. This study placed autophagy in the framework of the TME.

To summarise these studies, it should be noted that Rab4 and filamin-A affected the plasma membrane localisation of furin under hypoxic conditions. The plasma membrane localisation of furin under hypoxic conditions promoted cancer cell invasion [44]. CASC4 increased focal adhesion complexes but reduced the activity of Rho-GTPase Cdc42. It also suppressed tumour migration, while PC7 and furin cleaved CASC4 [45]. B3 decreased mature MT1-MMP and MMP-2 and inhibited invasiveness. Furin/PC7 was linked to tumour progression via MMP-2 activity on the extracellular matrix [47]. Autophagy could affect the TGN and tumour progression [27, 48].

## Therapeutic considerations and conclusions

Golgi proteins can be therapy targets. For example, ARF proteins and adaptors are crucial for transport from the TGN to the plasma membrane [49]. Brefeldin A inhibited the ARF1-GEF exchanger indicated in vesicle transport. Despite this, the Brefeldin A compound has not passed beyond the preclinical stage due to bioavailability issues. Instead, a study used another ARF1-GEF exchanger inhibitor, AMF-26. It inhibited ARF1 activation while inducing Golgi damage and cell death. AMF-26 affected the cis- and trans-Golgi, endosomes, and membrane trafficking. In xenograft mouse models, AMF-26 administration reduced tumour growth of BSY-1 breast cancer cells [49]. Other drugs were discussed; B3 inhibited furin and cancer motility [47]. Other studies have suggested a systematic approach to targeting the TGN [50]. Firstly, the stimulator of interferon genes (STING), an innate immune response, could be targeted. The STING pathway is highly dependent on vesicular trafficking, the ER and the TGN. Accordingly, defective CD8 + cell function and increased tumour growth were observed in STING mutant mice. Hence, modulating STING is a viable strategy [51]. Secondly, targeting glycosylation patterns in cancers is relevant, including detecting and targeting abnormal glycosylation marks deposited by the TGN and Golgi in cancers. For instance, blocking α-1,6 fucosyltransferase reduced invasion in cancer [52]. This was similar to the GOLPH3 knockdown study in which the post-translational modification of the EGFR was affected [29]. Finally, targeting trafficking is relevant. Since tumourigenesis and cancer progression rely on unconventional protein secretion profiles, it is possible to target them. One excellent example is the Golgi reassembly-stacking protein 55 kDa (GRASP55) involved in proliferation. It is upregulated following p53 mutation. Inhibiting this protein using a small molecule inhibitor, GRASPIN, suppressed tumour growth [53, 54]. Future efforts must focus on developing such ideas for cancer treatment. The conclusions of this study are as follows.

1. Rab proteins (i.e., Rab9 and Rab42) promote tumour progression by influencing trafficking and interacting with other proteins and pathways (e.g., p40, E2F1, immune tolerance, and checkpoint proteins). Rab21 cooperated with the retromer complex for the retrograde trafficking of cargo and promoted tumour proliferation [18, 20–23]. Also, Rab-related immune tolerance and cargo transport were linked by MIB2 (a ubiquitin ligase). MIB2 triggered PD-L1 trafficking to the cell surface via the TGN. MIB2 added K63-linked ubiquitin as a sorting signal to PD-L1, allowing its Rab8-mediated trafficking to the plasma membrane [55].
2. Golgi proteins affected inflammatory pathways and growth factor receptor dynamics. GOLM1 affected EGFR trafficking and promoted tumour progression [25]. GOLPH3 affected EGFR recycling and post-translational modifications and promoted tumour growth by triggering autophagy [27, 29]. Golgin-97 suppressed tumour progression by affecting NF-κB, ERK1/2 and p38 MAPK pathways [30, 32]. Giantin (a Golgi matrix protein) downregulation in breast cancer correlated with poor patient survival [56].
3. TGN-related proteins impacted tumour progression by modulating various signalling pathways. GCC2 promoted tumour growth by modulating vesicle trafficking of EGFR to the nucleus [33]. Syntaxin 6 promoted proliferation and invasion through the RACK1/STAT3 axis [34]. Other studies reported the axis of syntaxin 6/USF2/LC3B which promoted autophagy flux and tumour progression in HCC, showing that syntaxin 6 could trigger autophagy and affect the RACK1/STAT3 axis [57].
4. Clathrin-based endocytosis and TGN pumps affected growth factor receptor trafficking and, through that, tumour progression. AP-1 as an adaptor of clathrin, affected the cell membrane localisation of EGFR and proliferation [36]. Clathrin-based endocytosis route was also used for internalising FGFR4/FGF1 [38]. Further, SPCA1 was required for IGF1R formation [39]. Studies reported that 2-methylcoprophilinamide (a Golgi disruptor) and Brefeldin A suppressed the trafficking of RTK from the ER/TGN to the cell membrane, suggesting the role of the Golgi in growth factor receptor dynamics [58].
5. The links between the TGN, the TME, and tumour progression were dissected. Rab4 and filamin-A affected furin function and localisation under hypoxia and promoted tumour invasion [44]. CASC4 (a furin target) increased focal adhesion complexes and suppressed tumour migration [45]. B3 inhibited invasiveness [47]. Also, autophagy could promote tumour progression by mobilising nutrients from the TME [48]. Finally, hypoxia triggered Rab11 trafficking of integrin alpha6beta4 to the cell surface and stabilised microtubules, contributing to the invasive phenotype [59]. Understanding the link between the TGN components and tumour progression will improve treatment options.

## Data Availability

No datasets were generated or analysed during the current study.

## Abbreviations

AP-1: Clathrin adaptor complex-1
Arl1: Arf-like protein 1
CASC4: Cancer susceptibility candidate 4
CGN: Cis-Golgi networks
CHC: Clathrin heavy chain
CHO: Chinese hamster ovary
EGFR: Epidermal growth factor
ER: Endoplasmic reticulum
ERGIC: ER–Golgi intermediate compartment
FGFR: Fibroblast growth factor receptor
GAP: GTPase activating protein
GCC2: GRIP and coiled-coil protein 2
GEF: Guanine nucleotide exchange factor
GOLM1: Golgi membrane protein 1
GOLPH3: Golgi phosphoprotein 3
GRASP55: Golgi reassembly-stacking protein 55kDa
KO: Knockout
HCC: Hepatocellular carcinoma
IGF1R: Insulin-like growth factor 1 receptor
LC3-II: Microtubule-associated protein 1 light chain 3
MMP-2: Matrix metalloprotease 2
NHE7: Na + /H + transporter 7
PCNA: Proliferating cell nuclear antigen
PC7: Proprotein convertase 7
PD-L1: Programmed death-ligand 1
RTK: Receptor tyrosine kinase
SBP: Streptavidin-binding protein
SERCA2: Endoplasmic reticulum calcium ATPase
SNARE: Soluble N-ethylmaleimide sensitive factor attachment protein receptor
STAMP1: Six-transmembrane protein of prostate 1
STING: Stimulator of interferon genes
TGN: Trans-Golgi network
TME: Tumour microenvironment
VTC/S: Vesicular tubular complex/structure

## Declarations

### Declaration of the use of generative AI or AI-associated tools

No such tools were used.
